# Scalable Video Streaming Relay for Smart Mobile Devices in Wireless Networks

**DOI:** 10.1371/journal.pone.0167403

**Published:** 2016-12-01

**Authors:** Dongwoo Kwon, Huigwang Je, Hyeonwoo Kim, Hongtaek Ju, Donghyeok An

**Affiliations:** 1 Department of Computer Engineering, Keimyung University, Daegu, Korea; 2 Product R&D Division, Medical Device Development Center, Daegu, Korea; West Virginia University, UNITED STATES

## Abstract

Recently, smart mobile devices and wireless communication technologies such as WiFi, third generation (3G), and long-term evolution (LTE) have been rapidly deployed. Many smart mobile device users can access the Internet wirelessly, which has increased mobile traffic. In 2014, more than half of the mobile traffic around the world was devoted to satisfying the increased demand for the video streaming. In this paper, we propose a scalable video streaming relay scheme. Because many collisions degrade the scalability of video streaming, we first separate networks to prevent excessive contention between devices. In addition, the member device controls the video download rate in order to adapt to video playback. If the data are sufficiently buffered, the member device stops the download. If not, it requests additional video data. We implemented apps to evaluate the proposed scheme and conducted experiments with smart mobile devices. The results showed that our scheme improves the scalability of video streaming in a wireless local area network (WLAN).

## Introduction

With the advent of smart mobile devices and the development of wireless communication technology, mobile traffic is increasing dramatically. In addition, with the introduction of cyber physical systems (CPSs) to control and monitor physical objects with a software algorithm, mobile traffic is likely to further increases in the future [1]. Video traffic such as IPTV and YouTube already accounted for more than 50% of the total mobile traffic in 2014, and video traffic is expected to grow tenfold in 2019 [2]. With the growing market for smart mobile devices and increasing demand for video streaming, users want to be able to seamlessly watch videos on multiple smart mobile devices such as smartphones, tablets, and laptops. N-screen technology has emerged as a way to share multimedia content to multiple devices.

In a wireless local area network (WLAN), many smart mobile devices with N-screen technology connect to an access point (AP) to share video content. However, as the number of devices increases, many devices suffer from frequent playback interruptions and buffering during video streaming. Because all devices share the bandwidth of the wireless channel, the throughput of each smart device decreases as the number of smart devices increases [3–7].

The multicast approach has been proposed for efficient video streaming because it constructs a hierarchical network topology to reduce duplicate transmissions [8–10]. Despite this advantage, the deployment of multicast for the Internet has been limited [11, 12]. A peer-to-peer (P2P) based multicast scheme called ZIGZAG has been proposed [13]. However, the multicast scheme cannot be adapted directly for a WLAN because it requires a multi-hop connection between a source device and destination device to construct the tree topology. In a WLAN, because all devices are directly connected to an AP, constructing the tree topology requires additional overhead and degrades the performance.

**Fig 1:**
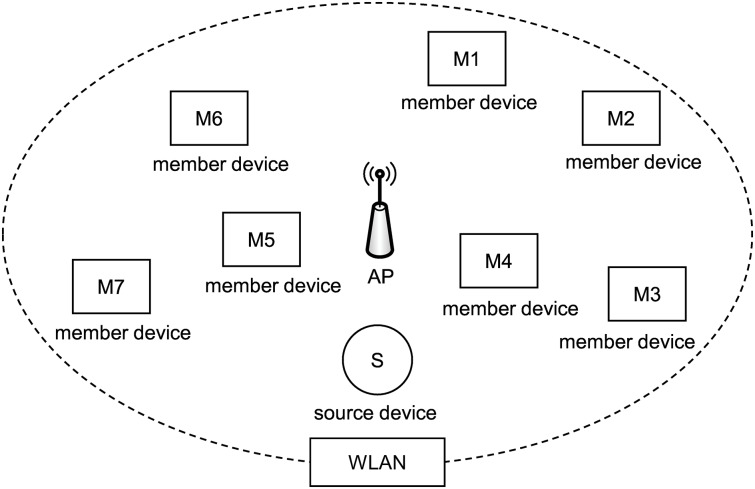
Example of video streaming in a WLAN.

**Fig 2:**
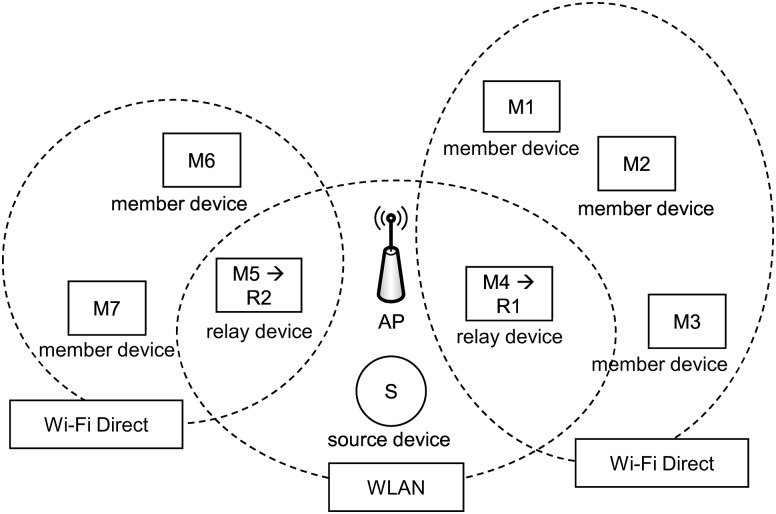
Example of video streaming with the proposed scheme.

In this study, we focused on the scalable video streaming technique for wireless networks. We exploited the relay node in order to extend the streaming service area and separate networks to reduce collisions among devices in a WLAN. Fig 1 presents an example of a video streaming service in a WLAN. Eight nodes, including one source node and seven member nodes, share the wireless channel. As the number of nodes increases, collision frequency increases, and the total throughput decreases. The throughput of each node is one-ninth of the performance of the WLAN. To resolve this problem, member nodes M4 and M5 act as relay nodes as shown in Fig 2. Because the three nodes (i.e., two relay nodes and one source node) contend over the wireless bandwidth of the WiFi Direct, the number of collisions decreases, and the throughput of each node increases. In the figure, M1, M2, and M3 communicate with the relay node R1 (i.e., member node M4) via WiFi Direct. Because the wireless channels of two WiFi Direct networks and one WLAN are different, the transmission in the WLAN does not interfere with the transmission in WiFi Direct. Because each network has three or four nodes, fewer collisions occur.

**Fig 3:**
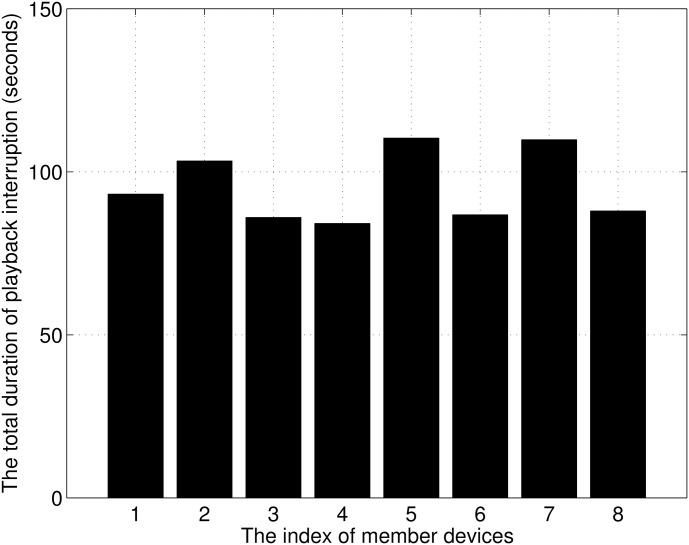
Total duration of playback interruption.

As shown in Fig 2, a source device that contains an original video file and relay devices are connected in a WLAN, and WiFi Direct technology is used to connect the relay device and member devices. With the progressive download scheme, which is popular for video streaming services, the streaming service is frequently interrupted by excessive contention. To observe the total duration of the playback interruption, we measured the playback interruption time for a WLAN. A source device transmitted a video file at about 4000 kbps to a relay device, and the relay device retransmitted it to the eight member devices. Fig 3 shows the results. All devices experienced playback interruption for 95 seconds on average.

**Fig 4:**
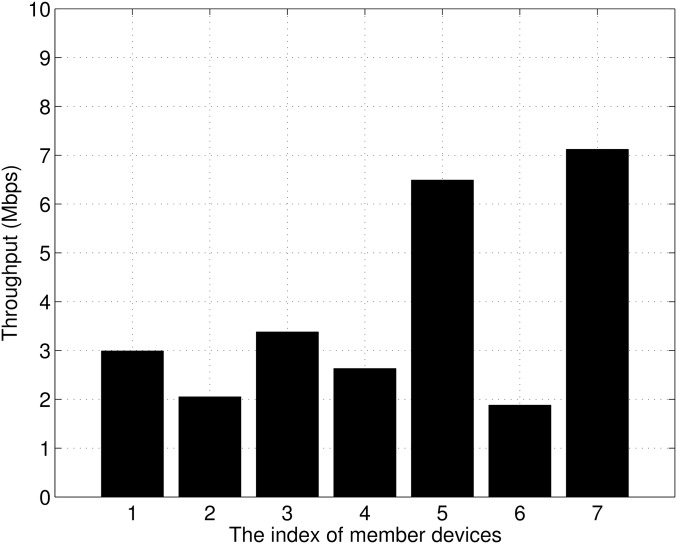
Measured throughput for each member device.

In the progressive download scheme, a whole video file is transmitted from the source device to the member device via the relay device until the download is completed. Therefore, the progressive download scheme causes many collisions between devices during the download, and video playback interruptions occur frequently. To observe the performance degradation due to contention between member devices, we measured the transmission control protocol (TCP) throughput in WiFi Direct networks. In the experiment, seven smart devices transmitted packets to a relay device, and Iperf was used to generate packets [14]. As shown in Fig 4, the average throughput was about 3.8 Mbps, and the throughput variation was large. Two devices (members 5 and 7) showed throughputs of greater than 6 Mbps, while the others had less than 4 Mbps. Because the bit rate of the video was 4000 kbps, playback interruption occurred in five of the member devices.

For wireless networks, several streaming relay schemes have been proposed to share multimedia content to multiple smart mobile devices [15–18]. Lin et al. proposed a payload-sharing mechanism to diminish the computation overhead of the relay device [15]. For scalable distribution, a large video file is divided into small files, and the multi-interface technique is utilized [16, 17]. In addition, the caching technique is used to improve efficiency [18]. However, network congestion still occurs in a WLAN. To provide seamless streaming, the streaming rate can be adjusted to the network bandwidth [19–21]. However, these approaches decrease the video resolution. In addition to streaming relay schemes, future Internet architecture called the content centric networking (CCN) has been proposed for efficient video distribution [22, 23].

In this paper, we propose a scalable video streaming relay scheme to decrease the contention between devices and maintain the video resolution. In the progressive download scheme, much of the video data that are not playing are stored in a player buffer, which results in excessive congestion in the WLAN. In our proposed scheme, a member device decides whether or not to continue the download. First, a member device that wants to use a video streaming service requests a portion of video (i.e., chunk) from a source device. The source device transmits the video data to a relay device, and the relay device forwards it to the member device. If the size of the buffered data is larger than the threshold, the member device stops the download. Otherwise, the member device requests and downloads an additional chunk file. After the chunk is downloaded, because the member device is excluded from contention, the contention overhead decreases, and the efficiency of the video streaming relay scheme is improved.

The rest of this paper is organized as follows. First, we present related works. We then present the proposed mechanism of the streaming relay scheme and architecture. We next discuss experiments performed to evaluate our scheme. Finally, we present our conclusions.

## Related Work

Many papers have proposed method to support a scalable and seamless video streaming service. “Scalable” refers to the number of smart devices that rarely experience playback interruption. “Seamless” means that the video is playing without a blank screen or playback interruption. Multicast has been widely adopted to realize efficient streaming service [8, 9]. However, deploying multicast on the Internet is difficult. To overcome this limit, Tran et al. proposed the P2P-based ZIGZAG to deliver video content to multiple video consumers [13]. ZIGZAG organizes a multicast tree by the logarithmic height to reduce the streaming delay between the content server and consumers. In a WLAN, however, because smart mobile devices connect to an AP directly, ZIGZAG based on a multicast scheme generates tree construction overhead. Thus it is not appropriate for video streaming in a WLAN. In addition to multicast, the Gray code improves the performance of distributed video coding. Song et al. conducted experiments and analyzed the results to determine how the Gray code enhances the performance [24].

In overlay multicast networks, an intermediate node with low system capability can become a bottleneck. This can cause a user to experience low-quality video streaming. To mitigate the computational burden of a bottleneck node, Lin et al. proposed one-to-many streaming splicing (OMSS) which utilizes a payload-sharing mechanism to improve the efficiency of memory copying and the worker-pool processing model for a multiprocessor [15]. However, OMSS has low backward compatibility because it requires kernel modification.

Hwang et al. proposed the enhanced adaptive fast replica (EAFR) based digital living network alliance (DLNA) proxy to distribute video content in home networks [16]. When the proxy receives a request from a user, it downloads the video file. The proxy divides the file into small files and then transmits the small files to the user. The user plays the downloaded files. However, because this scheme is based on a specific platform, the deployment is limited. Tawalbeh et al. introduced a mobile cloud computing (MCC) model based on Cloudlet to increase communication efficiency [25]. In this model, because the mobile devices communicate with MCC instead of a cloud server, the response time can be decreased. While technical difficulties should be addressed to deploy the MCC model, our proposed scheme can be deployed in smart devices.

In cellular networks, the network utilization varies over time. Because traffic jams occur during busy hours, a user experiences low throughput rather than the expected throughput. Keller et al. proposed MicroCast, which exploits both a cellular interface and wireless (or Bluetooth) interface to mitigate this problem [17]. Each smart device downloads a portion of the multimedia content and they share the downloaded data with nearby neighbors. MicroCast requires modification of the wireless driver and firmware of the smart device. In contrast to the above studies, our proposed scheme is designed to operate in the application layer without a driver or firmware update.

Bethanabhotla et al. proposed a scheduling policy to deal with the increased load of video on demand (VoD) in wireless networks [18]. In wireless networks, helper nodes are located at fixed positions and provide VoD stored in their cache to the client. However, when helpers move away from an AP without notification in a WLAN, clients suffer from playback interruption.

An optimal streaming strategy has been proposed to dynamically adjust the streaming rate over the Hypertext Transfer Protocol (HTTP) in wireless networks by utilizing a finite markov decision process (MDP) [19, 20]. However, this optimal streaming strategy investigates not the scalability but optimal streaming rate. Our proposed scheme focuses on scalability for video streaming in wireless networks.

Stockhammer proposed dynamic adaptive streaming over HTTP (DASH) to support videos with different levels of quality [21]. A client may decide on the proper video based on the network bandwidth, display resolution, etc. Because a content server should encode different video resolutions of a video before a client’s request with DASH, additional encoding overhead is required. In addition, calculating the appropriate video resolution is difficult.

As the demand for video streaming increases, a new Internet architecture called CCN has been proposed to efficiently transmit video files to end users [22, 23]. In CCN, the name of the video is used to route video files from a source node to an end user, and an intermediate router stores video content in its cache to reduce duplicate transmissions. Similar to CCN, a relay device stores the video content in the buffer. Nevertheless, because CCN is clean-slate architecture, the deployment of CCN on the Internet has been limited. In contrast, the proposed scheme can be implemented on commercial smart mobile devices.

Do et al. exploited both cellular and wireless networks for scalable video streaming [26]. They implemented and evaluated several algorithms on a real testbed. They considered scalable video streaming in cellular networks, but our proposed scheme improves the scalability of streaming in wireless networks.

As the popularity of IoT increases, a scalable security scheme has become necessary. Butun et al. proposed the cloud-centric multi-level authentication (CMULA) scheme to support the scalability of IoT [27]. A hierarchical structure is utilized to diminish the communication overhead for authentication. The CMULA scheme deals with the scalability of authentication, but our proposed scheme focuses on the scalability of streaming service.

For seamless service, routing protocols have been proposed to prolong the lifetime of wireless sensor networks or support the security of mobile ad hoc networks [28–31]. Relay nodes are elected by various algorithms such as the P-SEP and queen-bee algorithm, and other nodes act as the relay node in the next data transmission phase to reduce energy consumption. In contrast to these papers, we address the seamless streaming service in wireless networks.

With advances in video technology, recent multimedia content contain not only two-dimensional (2D) but also three-dimensional (3D) images. However, because quality measurement techniques for stereo images are not mature, subjective assessments are used. Yang et al. proposed an objective quality metric for stereo images [32]. Yang et al. also proposed a local stereo matching that utilizes motion flow to decrease errors in order to evaluate the disparity in stereo video [33]. In an Internet of Things environment, data are acquired by various devices, and a 3D image can be obtained with a stereo camera. However, adapting the configuration of the stereo camera is difficult. Lin et al. proposed a self-assessment model for that purpose [34]. However, in this paper, our target streaming service is not 3D image but 2D image.

The proposed scheme has the following features. First, the proposed scheme is easily deployed in smart devices without modification of Android because it is implemented in application layer. Second, our approach utilizes WiFi Direct to divide an wireless channel into two or three wireless channel to reduce collisions and increase throughput of each device. It leads to scalability improvement and seamless streaming service.

## Proposed Scheme

The details of the proposed scheme are presented here. We first present the streaming relay mechanism. Then we discuss the architecture and implementation for the proposed scheme.

### Streaming Relay Mechanism

**Fig 5:**
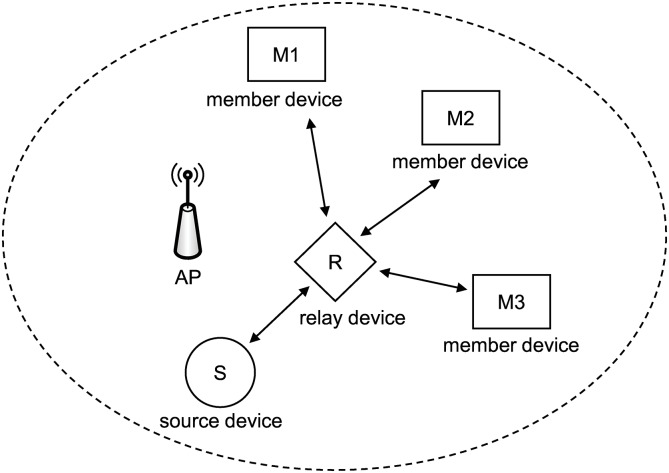
Streaming mechanism.

The proposed streaming scheme has two phases. Fig 5 illustrates the mechanism of the proposed streaming scheme. First, a member device requests video content from a source device via a relay device. Then, the relay device downloads the requested video content and stores the corresponding video file in the buffer. Second, a member device that is connected to the relay device through WiFi Direct requests and downloads a portion of the video file (i.e., chunk) for playing. After the requested chunk is downloaded, the member device checks the stored video data that are not playing in the buffer. If the stored video data are larger than a predefined value, the device stops to request the next chunk of the video until the size of the remaining video data in the buffer reaches the threshold. If not, the member device requests and downloads the next chunk immediately.

**Fig 6:**
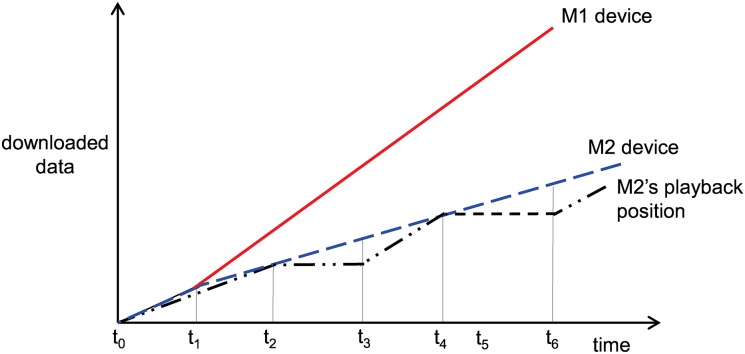
Example of devices M1 and M2 with the progressive download scheme.

**Fig 7:**
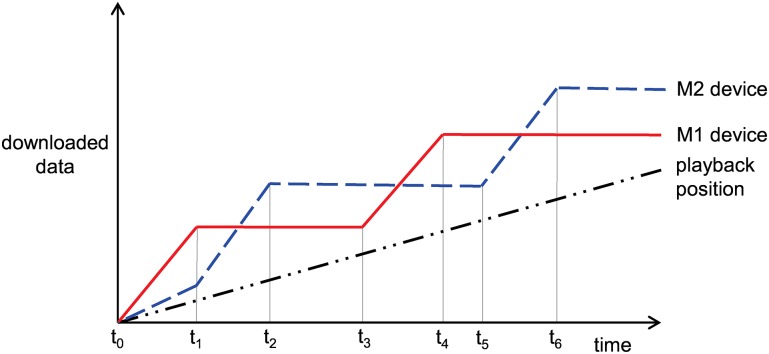
Example of devices M1 and M2 with the proposed scheme.

For example, both member devices M1 and M2 may request a video chunk file from the relay device, simultaneously. In the progressive download scheme, one of the two member devices can experience playback interruption. Fig 6 describes the download status and playback status of the member devices M1 and M2. The x- and y-axes represent the time and size of the downloaded file, respectively. The slopes of M1 and M2 represents the download rates from the relay device. M1 occupies more of the wireless channel than M2. At *t*_2_, the buffer of M2 is exhausted, and playback is interrupted until M2 downloads the next video chunk. At *t*_3_, playback resumes, but playback interruption occurs again. Our proposed scheme is shown in Fig 7. M1 wins the wireless channel contention and starts to download the video chunk file first. After the chunk is downloaded at *t*_1_, M1 stops the download and continues to play the video. At *t*_1_, M2 can download the requested chunk file faster because M1 stops downloading. Then, M2 stops to download the video chunk at *t*_2_. At *t*_3_, M1 realizes that the remaining data in the buffer is less than the threshold, so it initiates buffering for seamless video streaming. As the buffered data decrease, M2 also begins to download the next chunk at *t*_5_. Because M1 is idle, M2 can download the video file without contention and both devices can avoid inefficiency and unfairness due to channel contention.

When several member devices play video, each requests the video file with different resolutions because of different hardware specifications. We assume that a source device has several video files with different bit rates in order to support different resolutions. When a member device requests a video file from the source device, if the source device has a video file whose bit rate matches that of the requested video, it transmits the video chunk to the member device. If not, the source device sends a video chunk file with similar bit rate. For example, a source device has video files with bit rates of 1000, 2000, and 3000 kbps. If a member device requests a video with 1700 kbps, the source transmits the video chunk file with bit rate of 2000 kbps.

As the video resolution increases, the size of the video file also grows. Therefore, each member device requires different chunk sizes because of the different video resolutions. To determine the size of the next chunk to be sent, we assume that the total size of the video file, *S*_*total*_, and the total video playback duration, *T*_*total*_, are known. The size of the next chunk to be downloaded, *S*_*chunk*_, is calculated by

$$\begin{array}{c} S_{\text{chunk}}=\frac{S_{total}}{T_{total}}\;\times \;T_{range}\;\times \;\alpha \end{array}$$ (1)

where *T*_*range*_ is the minimum playback time and *α* is a constant value to guarantee the minimum playback time. For example, when a member device requires a chunk that can be played for 10 seconds, *T*_*range*_ is set to 10. However, *T*_*range*_ does not guarantee the playback duration because the video resolution is an average value. Each scene has a different file size. If the screen changes quickly, the file size of the scene is large. Otherwise, the size of the scene decreases. To deal with the variable length of the chunk, we use the constant value of *α*. In this paper, we set *α* to 2, and this is discussed in more detail in the subsection “Variable Chunk Size”.

In the proposed scheme, a source device is determined depending on its own video files. For instance, two devices A and B have video file A and B, respectively. If the video A is required for streaming, the device A acts as the source device because it has the video file A. The device B becomes the relay or member device, and it requests the chunk of the video A from the source device (i.e., device A). Additionally, if the device A has two different videos A and B, it becomes the source device for video A and B. In this case, each member device plays different video. It means that our proposed scheme is able to support P2P like video streaming service because different video files stored in different devices can be transmitted each other.

Algorithm 1 presents the pseudo-code for the proposed streaming scheme. If the size of remaining video data in the buffer is less than the threshold, the member device transmits the next video chunk request to relay (or source device). If the relay (or source device) receives the chunk request from the member device (or the relay device) and has the video chunk, it sends the corresponding chunk to the member device (or the relay device). Besides, source, relay, and member devices try to keep playing the video if they have video data in the buffer. This algorithm is repeated until playing all video chunks is finished. Therefore, the time complexity of this algorithm O(n) if the *n* is the number of chunks for the video.

**Algorithm 1** Algorithm for the proposed streaming scheme

*S*_*total*_ ← the total size of the video file

*T*_*total*_ ← the total video playback duration

*T*_*range*_ ← the minimum playback time

*alpha* ← 2

$S_{chunk}=\frac{S_{total}}{T_{total}}\;\times \;T_{range}\;\times \;\alpha$

*C*_*n*_ ← 0, as the current chunk number for playing the video

*P*_*next*_ ← 0, as the next chunk number for download

$C_{total}\leftarrow \frac{S_{total}}{S_{chunk}}$, as the total number of the chunks

**if** *S*_*total*_ mod *S*_*chunk*_ ≠ 0 **then**

 *C*_*total*_ ← *C*_*total*_+1

**end if**

**while** *C*_*n*_ < *C*_*total*_ **do**

 **if** *P*_*next*_ < *C*_*total*_ and VideoPlayer.RemainingBuffer ≤ threshold **then**

  transmit the next chunk request *C*_*req*_ to the source or relay device

  store the received video chunk *P*_*next*_ in the video player’s buffer

  *P*_*next*_ ← *P*_*next*_+1

 **end if**

 **if** the device receives a chunk request for *C*_*res*_ and *C*_*res*_ < *P*_*next*_ **then**

  transmit the video chunk *C*_*res*_ to the relay or member device

 **end if**

 **if** the video chunk *C*_*n*_ is not playing **then**

  start to play the video chunk *C*_*n*_

 **else if** the video chunk *C*_*n*_ play is finished **then**

  *C*_*n*_ ← *C*_*n*_+1

 **else**

  continue to play the video chunk *C*_*n*_

 **end if**

**end while**

### Architecture and Implementation

**Fig 8:**
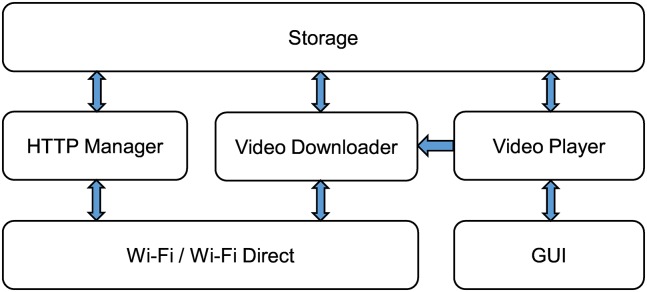
Architecture of the relay device.

To support scalable video streaming, we present the following architecture of the relay and member devices. Fig 8 presents the architecture of the relay device. The relay device consists of three main components: (1) A video downloader that requests the chunk and stores the video file once the remaining data in the buffer decrease under the threshold, (2) a video player that reads and plays the video file in storage, and (3) an HTTP manager that receives the video chunk request from a member device and transmits the corresponding video chunk to the member device.

**Fig 9:**
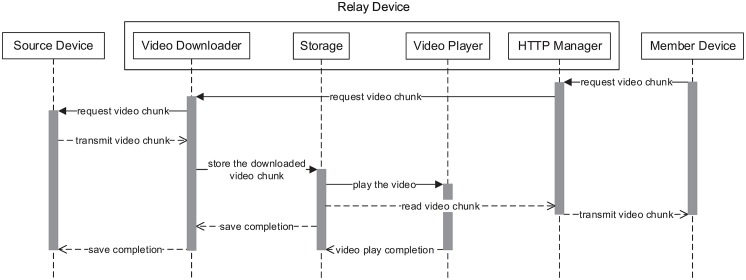
Sequence diagram of the relay device.

Fig 9 shows the sequence diagram of the relay device. First, the member device requests a video chunk that is not stored in the relay device. The relay device requests the video chunk from the source device, and the video chunk is stored. After the download finished, the relay device plays the video. Then, the HTTP manager reads the video chunk and sends it to the member device.

**Fig 10:**
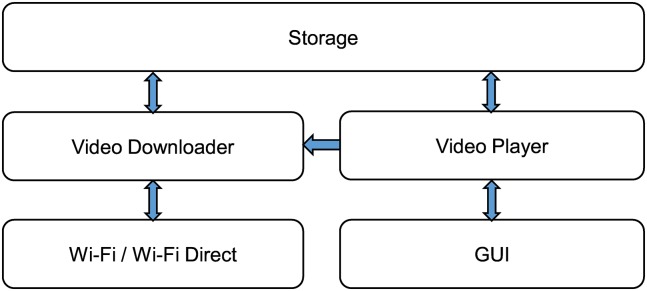
Architecture of the member device.

**Fig 11:**
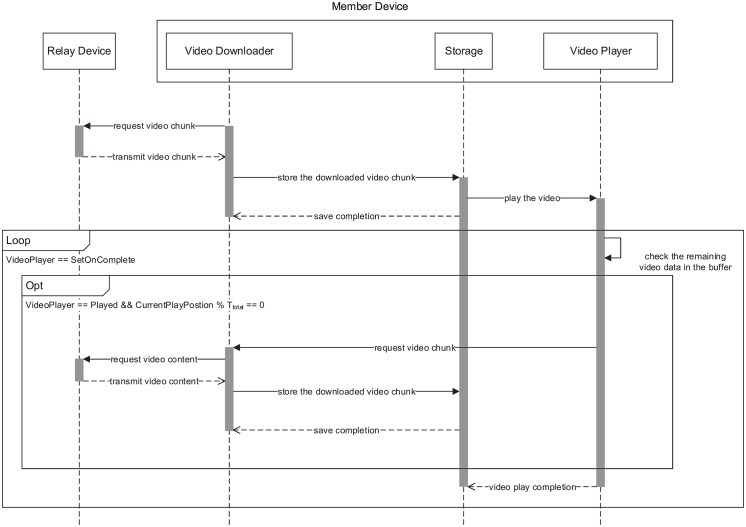
Sequence diagram of the member device.

Fig 10 illustrates the architecture of the member device. Unlike the relay device, because the member device is aiming to download and play the video, it consists of two components: the video downloader and video player. The video downloader is responsible for downloading the video from the relay device. The video player plays the downloaded video. Fig 11 presents the sequence diagram of the member device. To play the video, the video downloader requests the video from the relay device and stores the received video chunk. Then, the video player plays the video and periodically checks its buffer as it is playing. If the saved video data are less than the threshold, the video player makes the video downloader download the next video chunk.

**Fig 12:**
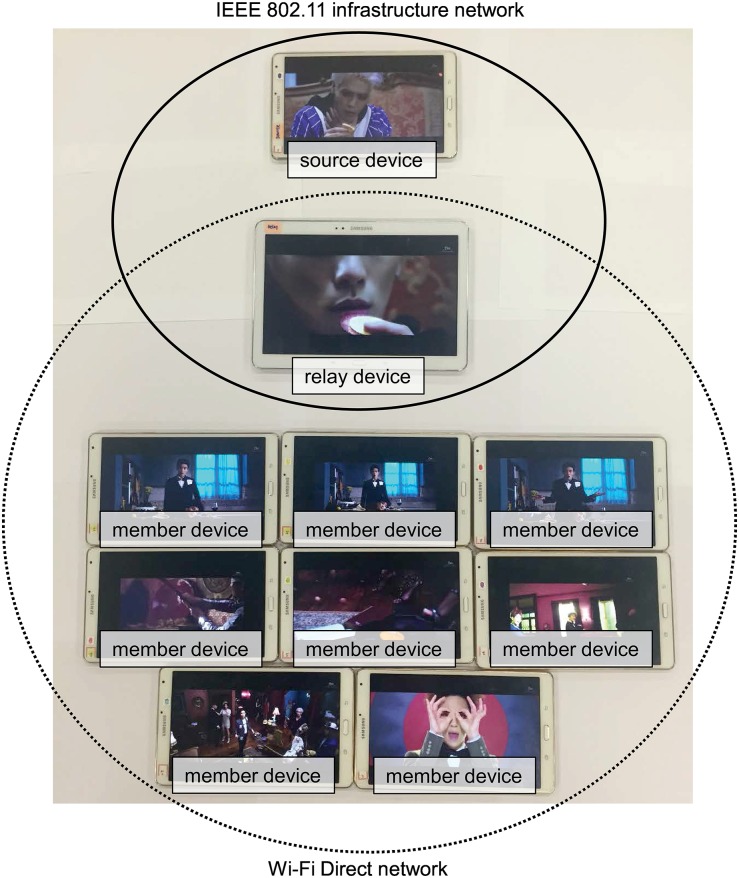
Snapshot of the experiment.

Fig 12 shows the video streaming relay. We implemented the proposed scheme on an Android device with a level 19 application programming interface (API). Both the relay and member devices were implemented based on the architecture in Figs 8 and 10. The relay device has two connections. The first is the connection between the source and relay devices via the AP in the WLAN. Each member device connects to the relay device via WiFi Direct [35]. The video player is implemented with VideoView, which is provided by Android OS. The video downloader utilizes an HTTP request/response pair to request and download a video chunk and uses the RamdomAccessFile class in Java to store the video chunk. The HTTP manager is implemented by using NanoHTTPD, which is a light-weight HTTP server [36].

## Performance Evaluation

We evaluated the performance of the proposed scheme by comparing its results with those of the progressive download scheme that transmits the whole video file to the member device [19, 37]. As discussed in the subsection “Architecture and Implementation”, we implemented a video streaming relay scheme on an Android device for the performance evaluation. First, we measured the variable length of a chunk to determine *α* in Eq (1). Then, we measured the number of playback interruptions and the playback interruption ratio when the video was playing. Finally, we performed an experiment to measure the maximum number of member nodes that can play a video without playback interruption.

### Variable Chunk Size

A video consists of many chunks, and the proposed scheme downloads a chunk for video playback. When a member device downloads chunks of equal size, it experiences different playback durations for each chunk because each scene has a different video resolution. Therefore, the member node downloads a larger chunk size to guarantee the playback duration.

To determine *α* in Eq (1), we conducted measurements and varied *α* from 1 to 3. In the measurements, *T*_*range*_ of Eq (1) was set to 10. Table 1 presents the video specifications. Table 2 presents the results. When the value of *α* is set to 1, four of the five videos played about 8-9 seconds. This means that the playback duration of the downloaded video chunk was less than expected. As indicated in Table 2, the playback duration tended to increase with *α*. Then, the playback duration of the chunk became larger than the expected duration, *T*_*range*_. However, when *α* was large, because the length of the chunk also increased, it caused unnecessary downloading. Based on the measurement results, we set *α* to 2.

**Table 1:** Video specification used in the measurement.

	video 1	video 2	video 3	video 4	video 5
Bit rate	2692 Kbps	2662 Kbps	4245 Kbps	2645 Kbps	3148 Kbps
Video format	MPEG-4	MPEG-4	MPEG-4	MPEG-4	MPEG-4
File size	57.3 MB	107 MB	110 MB	92.6 MB	525 MB
Duration	178 seconds	336 seconds	217 seconds	293 seconds	1399 seconds

**Table 2:** Playback duration with 10 of *T*_*range*_.

*α*	video 1	video 2	video 3	video 4	video 5
1	9.64 seconds	8.72 seconds	7.98 seconds	10.11 seconds	8.94 seconds
2	17.03 seconds	19.06 seconds	17.90 seconds	18.12 seconds	16.98 seconds
3	26.67 seconds	28.88 seconds	29.25 seconds	27.52 seconds	28.49 seconds

### Playback Interruption

**Fig 13:**
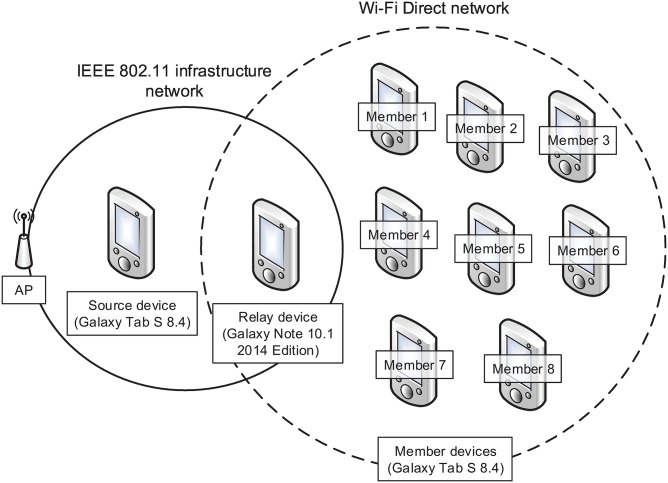
Network topology in the experiment.

We evaluated the performance of the proposed scheme. Fig 13 shows the experiment topology. A source device and relay device were connected to an AP directly, and eight member devices communicated with the relay device through WiFi Direct. Experiments were performed with a Galaxy Note and nine Galaxy Tab devices. Table 3 lists the hardware specifications and OS version. The Android devices were located at Keimyung University (KMU), Daegu, South Korea. When a video was played, we measured the number of playback interruptions. Then, we observed the playback interruption ratio. We used an MPEG-4 video with a playback duration of 227 seconds, and encoded the video at four different bit rates: 2625, 3120, 3609, and 4101 kbps. Table 4 presents the video specifications.

**Table 3:** Hardware specification and OS version of Android devices.

	Galaxy Note 10.1	Galaxy Tab S 8.4
CPU	1.9 GHz (Octa-Core)	1.3 GHz (Octa-Core)
RAM	3 GB	3 GB
WiFi	802.11a/b/g/n/ac	802.11a/b/g/n/ac
OS	Android 4.4.2 KitKat	Android 5.0.2 Lollipop

**Table 4:** Specification of the video in the experiment.

Playback time	227 seconds			
Bit rate	2625 Kbps	3120 Kbps	3609 Kbps	4101 Kbps
File size	71.1 MB	84.5 MB	97.8 MB	111 MB

#### Number of playback interruptions

**Fig 14:**
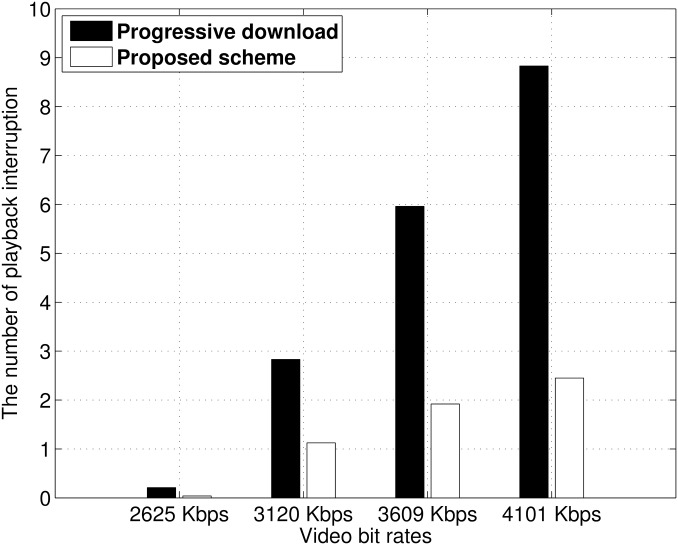
Average number of playback interruptions.

We measured the total number of playback interruptions when a video was played. We compared the results to those of the progressive download scheme, which has already been implemented in many streaming services [37]. Fig 14 shows the average number of playback interruptions of the member devices. As the video bit rate increased, the video file size also increased. Thus, the member devices experienced more frequent playback interruptions from increased contention. Our proposed scheme showed fewer playback interruptions than the progressive download scheme. When a video with a 4101 kbps bit rate was streaming, our streaming relay scheme achieved an approximately 70% reduction in interruptions compared to the progressive download. The difference between the proposed scheme and progressive download is as follows. The proposed scheme repeats a video chunk download cycle and idle cycle. After a video chunk is received, the member device changes from a download cycle to an idle cycle; therefore, the channel contention between member devices decreases. However, the progressive download scheme continues to download the whole video file, so some member devices experience starvation from high channel contention.

#### Playback interruption ratio

**Fig 15:**
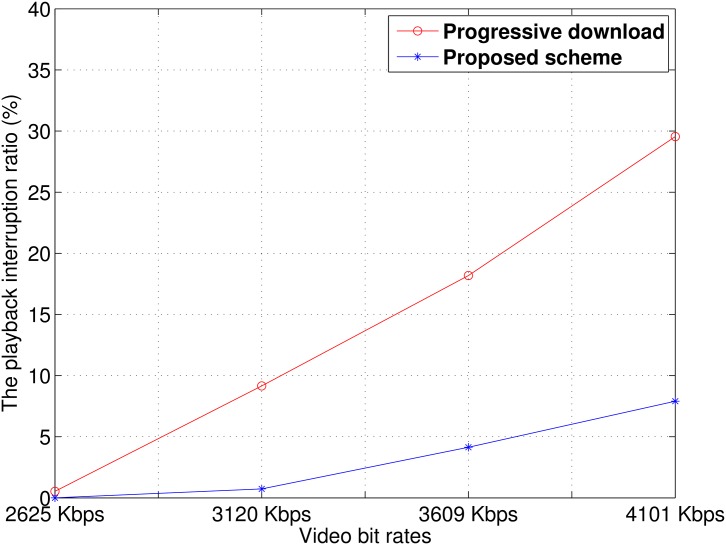
Playback interruption ratio.

We observed the playback interruption ratio. We measured the playback duration without interruption and the duration of playback interruptions. Then, as given by Xing et al. in [19], the playback interruption ratio, *R*_*PI*_, can be calculated as follows:

$$\begin{array}{c} R_{\text{PI}}=\frac{T_{I}}{T_{P}+T_{I}} \end{array}$$ (2)

where *T*_*P*_ and *T*_*I*_ are the durations of the playback and playback interruptions, respectively. A high playback interruption ratio indicates that member devices suffered from frequent playback interruption, but a zero playback interruption ratio means that the video could be played seamlessly. Fig 15 presents the results. As the video bit rate increased, the video file size also increased. With the progressive download scheme, the transmission of larger video files caused high channel contention between member devices. Therefore, playback interruptions occurred more frequently. With the proposed scheme, however, because member nodes in an idle cycle did not request the next video chunk, member devices in the download cycle experienced less contention than with the progressive download scheme.

**Fig 16:**
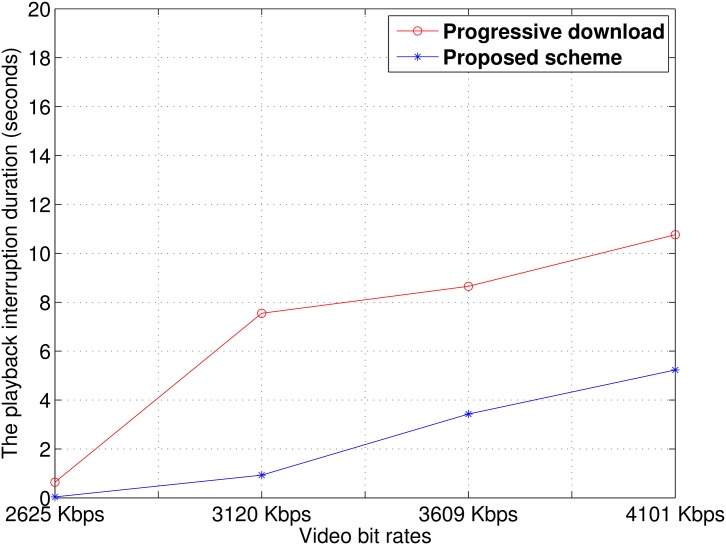
Average playback interruption duration.

We measured the duration of the playback interruption experienced by a member device. Fig 16 presents the average duration of the playback interruption for each video bit rate. As the video bit rate increased, the average playback interruption of both the progressive download and proposed scheme increased. With the progressive download, the duration of the playback interruption rapidly increased when the video bit rate was changed from 2625 kbps to 3120 kbps. This indicates that the wireless network was already saturated by excessive channel contention. In contrast, because our streaming relay scheme avoids excessive downloading from the relay device, the duration of the playback interruption was shorter than with the progressive download.

**Fig 17:**
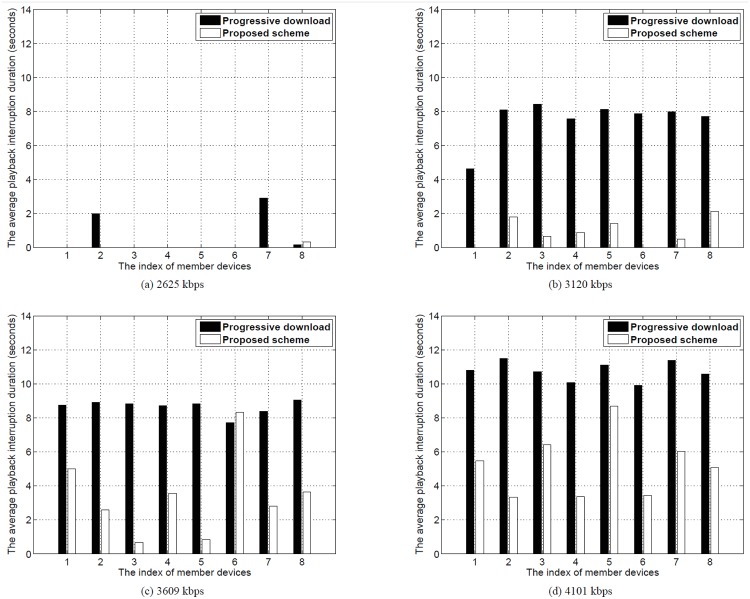
Average playback interruption duration for each member device. At (a) 2625 kbps, (b) 3120 kbps, (c) 3609 kbps, and (d) 4101 kbps.

Fig 17 shows the average playback interruption duration for each member device when a user watched a video with bit rates of 2625, 3120, 3609, and 4101 kbps. The proposed relay scheme showed a shorter interruption duration than the progressive download. As shown in Fig 17(a), playback interruption rarely occurred in both schemes. When the video at 3120 kbps was streaming, as shown in Fig 17(b), all devices with the progressive download scheme experienced a long playback interruption, but the video playback stopped for only a short time with our proposed relay scheme. With the proposed scheme, as the video bit rate increased, some devices struggled with other devices for video transmission, which resulted in frequent playback interruption. Despite the increased playback interruption, the video interruption duration of our proposed scheme was still less than that of the progressive download scheme.

### Scalability

**Fig 18:**
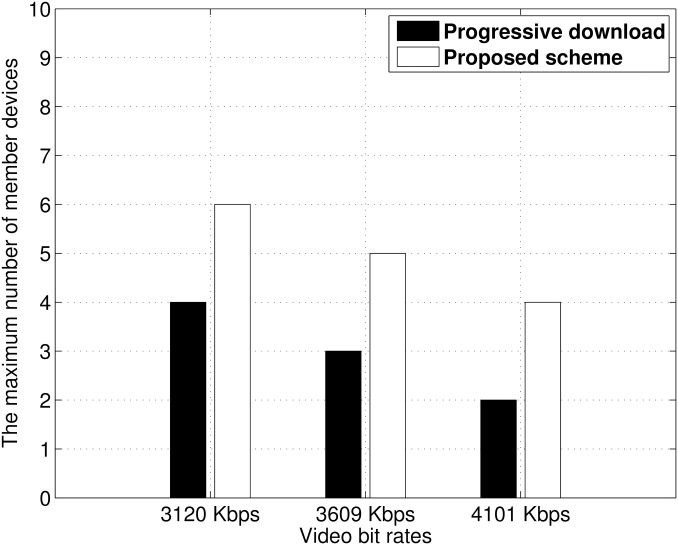
Maximum number of member devices when all play the video without interruption.

We evaluated the scalability of both the proposed scheme and progressive download scheme. We counted the maximum number of member devices that could provide streaming service without playback interruption. Fig 18 presents the results. For both the proposed scheme and progressive download scheme, the maximum number of member devices decreased with an increasing bit rate because of increased contention. However, the proposed scheme supported at least 50% more than the maximum number of member nodes with the progressive download scheme. In addition, the gap between both schemes stayed constant as the bit rate increases. This means that our replay scheme provides higher scalability than the progressive download scheme.

## Conclusion

We have proposed a scalable streaming relay scheme for wireless networks. The main advantages of our proposed scheme are follows. First, to decrease network congestion, a member device decides whether or not to download a video file. Second, our proposed scheme is designed for the application layer, so it does not require driver or firmware updates. Finally, we evaluated the performance through experiments on Android devices. Compared to the progressive download scheme, the proposed streaming relay scheme showed better scalability.

## Supporting Information

S1 Table Raw data of playback interruption measurement with the progressive download scheme (PDF file). (PDF)

S2 Table Raw data of playback interruption measurement with the proposed scheme (PDF file). (PDF)

S3 Table Results of network bandwidth measurement (PDF file). (PDF)

S4 Table Maximum number of devices without playback interruption (PDF file). (PDF)
