# Supplementary material for: Scalable Video Streaming Relay for Smart Mobile Devices in Wireless Networks
Source: PLoS One. 2016 Dec 1;11(12):e0167403. doi: 10.1371/journal.pone.0167403 (PMC5132399; doi:10.1371/journal.pone.0167403)
Supplement: S1 Table — (PDF) [file pone.0167403.s001.pdf]

2625 kbps

case 1	number o	average d	total duration
device 1	0.00	0.00	0.00
device 2	2.00	6.00	12.00
device 3	0.00	0.00	0.00
device 4	0.00	0.00	0.00
device 5	0.00	0.00	0.00
device 6	0.00	0.00	0.00
device 7	2.00	8.75	16.50
device 8	0.00	0.00	0.00

case 2	number o	average d	total duration
device 1	0.00	0.00	0.00
device 2	0.00	0.00	0.00
device 3	0.00	0.00	0.00
device 4	0.00	0.00	0.00
device 5	0.00	0.00	0.00
device 6	0.00	0.00	0.00
device 7	0.00	0.00	0.00
device 8	0.00	0.00	0.00

case 3	number o	average d	total duration
device 1	0.00	0.00	0.00
device 2	0.00	0.00	0.00
device 3	0.00	0.00	0.00
device 4	0.00	0.00	0.00
device 5	0.00	0.00	0.00
device 6	0.00	0.00	0.00
device 7	0.00	0.00	0.00
device 8	1.00	0.50	0.50

3120 kbps

case 1	number o	average d	total duration
device 1	2.00	5.75	11.50
device 2	5.00	8.60	43.00
device 3	2.00	7.25	14.50
device 4	1.00	6.00	6.00
device 5	5.00	7.40	37.00
device 6	1.00	8.00	8.00
device 7	1.00	7.50	7.50
device 8	1.00	7.00	7.00

case 2	number o	average d	total duration
device 1	5.00	8.10	40.50
device 2	6.00	6.67	40.02
device 3	4.00	10.83	43.32
device 4	4.00	8.75	35.00
device 5	4.00	8.75	35.00
device 6	4.00	8.13	32.50
device 7	4.00	8.63	34.50
device 8	4.00	8.88	35.50

case 3	number o	average d	total duration
device 1	0.00	0.00	0.00
device 2	2.00	9.00	18.00
device 3	2.00	7.25	14.50
device 4	2.00	8.00	16.00
device 5	2.00	8.25	16.50
device 6	2.00	7.50	15.00
device 7	3.00	7.83	23.50
device 8	2.00	7.25	14.50

3609 kbps

case 1	number o	average d	total duration
device 1	7.00	9.14	64.00
device 2	8.00	9.00	72.00
device 3	8.00	8.00	64.00
device 4	7.00	9.29	65.00
device 5	7.00	9.00	63.00
device 6	6.00	9.33	56.00
device 7	7.00	9.29	65.00
device 8	7.00	9.00	63.00

case 2	number o	average d	total duration
device 1	4.00	8.00	32.00
device 2	7.00	8.57	60.00
device 3	4.00	9.25	37.00
device 4	5.00	8.10	40.50
device 5	5.00	8.10	40.50
device 6	4.00	8.75	35.00
device 7	4.00	10.88	43.50
device 8	4.00	8.63	34.50

case 3	number o	average d	total duration
device 1	5.00	9.10	45.50
device 2	6.00	9.17	55.00
device 3	5.00	9.20	46.00
device 4	5.00	8.80	44.00
device 5	5.00	9.40	47.00
device 6	9.00	5.06	45.50
device 7	9.00	5.00	45.00
device 8	5.00	9.50	47.50

4101 kbps

case 1	number o	average d	total duration
device 1	6.00	10.58	63.50
device 2	9.00	11.44	103.00
device 3	6.00	10.42	62.50
device 4	6.00	10.17	61.00
device 5	10.00	10.55	105.50
device 6	7.00	8.71	61.00
device 7	9.00	12.33	111.00
device 8	6.00	10.75	64.50

case 2	number o	average d	total duration
device 1	9.00	12.17	109.50
device 2	9.00	12.33	111.00
device 3	9.00	10.67	96.00
device 4	9.00	9.28	83.50
device 5	9.00	12.39	111.50
device 6	9.00	10.44	94.00
device 7	10.00	10.40	104.00
device 8	9.00	10.00	90.00

case 3	number o	average d	total duration
device 1	11.00	9.68	106.50
device 2	9.00	10.67	96.00
device 3	9.00	11.06	99.50
device 4	10.00	10.80	108.00
device 5	11.00	10.36	114.00
device 6	10.00	10.55	105.50
device 7	10.00	11.45	114.50
device 8	10.00	10.95	109.50
