# Supplementary material for: Scalable Video Streaming Relay for Smart Mobile Devices in Wireless Networks
Source: PLoS One. 2016 Dec 1;11(12):e0167403. doi: 10.1371/journal.pone.0167403 (PMC5132399; doi:10.1371/journal.pone.0167403)
Supplement: S2 Table — (PDF) [file pone.0167403.s002.pdf]

2625 kbps

case 1	number o	average d	total duration
device 1	0.00	0.00	0.00
device 2	0.00	0.00	0.00
device 3	0.00	0.00	0.00
device 4	0.00	0.00	0.00
device 5	0.00	0.00	0.00
device 6	0.00	0.00	0.00
device 7	0.00	0.00	0.00
device 8	1.00	0.50	0.50

case 2	number o	average d	total duration
device 1	0.00	0.00	0.00
device 2	0.00	0.00	0.00
device 3	0.00	0.00	0.00
device 4	0.00	0.00	0.00
device 5	0.00	0.00	0.00
device 6	0.00	0.00	0.00
device 7	0.00	0.00	0.00
device 8	0.00	0.00	0.00

case 3	number o	average d	total duration
device 1	0.00	0.00	0.00
device 2	0.00	0.00	0.00
device 3	0.00	0.00	0.00
device 4	0.00	0.00	0.00
device 5	0.00	0.00	0.00
device 6	0.00	0.00	0.00
device 7	0.00	0.00	0.00
device 8	0.00	0.00	0.00

3120 kbps

case 1	number o	average d	total duration
device 1	0.00	0.00	0.00
device 2	2.00	1.25	2.50
device 3	2.00	0.50	1.00
device 4	0.00	0.00	0.00
device 5	1.00	1.50	1.50
device 6	0.00	0.00	0.00
device 7	0.00	0.00	0.00
device 8	2.00	3.50	7.00

case 2	number o	average d	total duration
device 1	0.00	0.00	0.00
device 2	1.00	2.00	2.00
device 3	1.00	1.00	1.00
device 4	2.00	1.50	3.00
device 5	1.00	1.00	1.00
device 6	0.00	0.00	0.00
device 7	1.00	1.00	1.00
device 8	0.00	0.00	0.00

case 3	number o	average d	total duration
device 1	0.00	0.00	0.00
device 2	3.00	2.17	6.50
device 3	1.00	0.50	0.50
device 4	3.00	1.17	3.50
device 5	2.00	1.75	3.50
device 6	0.00	0.00	0.00
device 7	1.00	0.50	0.50
device 8	2.00	0.75	1.50

3609 kbps

case 1	number o	average d	total duration
device 1	2.00	13.50	27.00
device 2	2.00	6.50	13.00
device 3	2.00	2.00	4.00
device 4	3.00	3.17	9.50
device 5	3.00	2.00	6.00
device 6	4.00	9.00	36.00
device 7	5.00	7.80	39.00
device 8	4.00	9.13	36.50

case 2	number o	average d	total duration
device 1	1.00	1.00	1.00
device 2	2.00	0.75	1.50
device 3	0.00	0.00	0.00
device 4	0.00	0.00	0.00
device 5	0.00	0.00	0.00
device 6	1.00	0.50	0.50
device 7	3.00	0.67	2.00
device 8	3.00	0.83	2.50

case 3	number o	average d	total duration
device 1	2.00	0.50	1.00
device 2	2.00	0.50	1.00
device 3	0.00	0.00	0.00
device 4	3.00	7.50	22.50
device 5	1.00	0.50	0.50
device 6	2.00	15.50	31.00
device 7	0.00	0.00	0.00
device 8	1.00	1.00	1.00

4101 kbps

case 1	number o	average d	total duration
device 1	1.00	2.00	2.00
device 2	1.00	2.50	2.50
device 3	2.00	4.00	8.00
device 4	1.00	2.50	2.50
device 5	0.00	0.00	0.00
device 6	3.00	2.83	8.50
device 7	0.00	0.00	0.00
device 8	2.00	2.25	4.50

case 2	number o	average d	total duration
device 1	0.00	0.00	0.00
device 2	3.00	3.00	9.00
device 3	1.00	0.50	0.50
device 4	1.00	0.50	0.50
device 5	2.00	6.75	13.50
device 6	0.00	0.00	0.00
device 7	1.00	0.50	0.50
device 8	0.00	0.00	0.00

case 3	number o	average d	total duration
device 1	5.00	22.50	112.50
device 2	5.00	0.20	1.00
device 3	5.00	13.70	68.50
device 4	6.00	5.67	34.00
device 5	3.00	27.67	83.00
device 6	6.00	5.42	32.50
device 7	3.00	17.00	51.00
device 8	2.00	6.75	13.50
