# Supplementary material for: Scalable Video Streaming Relay for Smart Mobile Devices in Wireless Networks
Source: PLoS One. 2016 Dec 1;11(12):e0167403. doi: 10.1371/journal.pone.0167403 (PMC5132399; doi:10.1371/journal.pone.0167403)
Supplement: S3 Table — (PDF) [file pone.0167403.s003.pdf]

	throughput
device 1	2.99
device 2	2.05
device 3	3.38
device 4	2.63
device 5	6.49
device 6	1.88
device 7	7.12
