# Supplementary material for: Scalable Video Streaming Relay for Smart Mobile Devices in Wireless Networks
Source: PLoS One. 2016 Dec 1;11(12):e0167403. doi: 10.1371/journal.pone.0167403 (PMC5132399; doi:10.1371/journal.pone.0167403)
Supplement: S4 Table — (PDF) [file pone.0167403.s004.pdf]

	3120 kbps	3609 kbps	4101 kbps
progressiv	4	3	2
proposed	6	5	4
